# A variant of ASIC2 mediates sodium retention in nephrotic syndrome

**DOI:** 10.1172/jci.insight.148588

**Published:** 2021-08-09

**Authors:** Marc Fila, Ali Sassi, Gaëlle Brideau, Lydie Cheval, Luciana Morla, Pascal Houillier, Christine Walter, Michel Gennaoui, Laure Collignon, Mathilde Keck, Gabrielle Planelles, Naziha Bakouh, Michel Peuchmaur, Georges Deschênes, Ignacio Anegon, Séverine Remy, Bruno Vogt, Gilles Crambert, Alain Doucet

**Affiliations:** 1 Cordeliers Research Center, Sorbonne University, INSERM, University of Paris, Laboratory of Renal Metabolism and Physiology, Paris, France.; 2 CNRS, ERL8228, Paris, France.; 3 Cytology and Pathology Department and; 4 Pediatric Nephrology Department, Robert Debré Hospital, Paris, France.; 5 INSERM UMR 1064, Center for Research in Transplantation and Immunology Transgenic Rats ImmunoPhenomic Facility, Nantes, France.; 6 Department of Nephrology and Hypertension, Inselspital, Bern University Hospital, Bern, Switzerland.

**Keywords:** Nephrology, Homeostasis, Ion channels, Sodium channels

## Abstract

Idiopathic nephrotic syndrome (INS) is characterized by proteinuria and renal sodium retention leading to edema. This sodium retention is usually attributed to epithelial sodium channel (ENaC) activation after plasma aldosterone increase. However, most nephrotic patients show normal aldosterone levels. Using a corticosteroid-clamped (CC) rat model of INS (CC-PAN), we showed that the observed electrogenic and amiloride-sensitive Na retention could not be attributed to ENaC. We then identified a truncated variant of acid-sensing ion channel 2b (ASIC2b) that induced sustained acid-stimulated sodium currents when coexpressed with ASIC2a. Interestingly, CC-PAN nephrotic ASIC2b-null rats did not develop sodium retention. We finally showed that the expression of the truncated ASIC2b in the kidney was dependent on the presence of albumin in the tubule lumen and activation of ERK in renal cells. Finally, the presence of *ASIC2* mRNA was also detected in kidney biopsies from patients with INS but not in any of the patients with other renal diseases. We have therefore identified a variant of ASIC2b responsible for the renal Na retention in the pathological context of INS.

## Graphical abstract

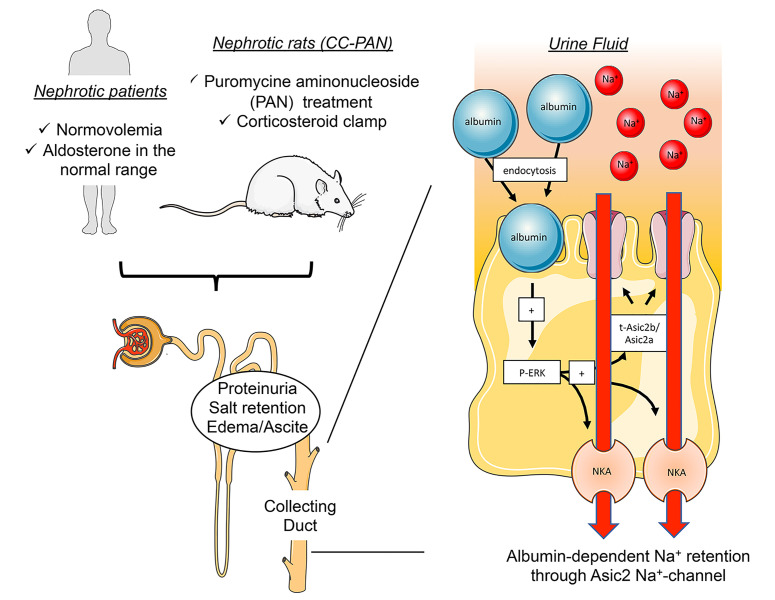

## Introduction

Nephrotic syndrome is defined by the association of massive proteinuria with hypoalbuminemia brought about by dysfunctions of the glomerular filtration barrier. Glomerular dysfunctions stem either from poorly characterized immunological disorders or from genetic alterations of glomerular proteins. Whatever its origin, idiopathic nephrotic syndrome (INS) is associated with sodium retention, which participates in the formation of ascites and edema (1). The site and mechanism of sodium retention have been deciphered using the puromycin aminonucleoside–induced rat model of nephrotic syndrome (PAN rat) that reproduces most biological and clinical signs of the human disease (2, 3). Sodium retention in PAN rats originates from the aldosterone-sensitive distal nephron (ASDN) and stems from the marked stimulation of the basolateral Na^+^/K^+^-ATPase and of the apical sodium channel epithelial sodium channel (ENaC), 2 molecular targets of aldosterone (4–6).

However, conversely to PAN rats most nephrotic patients display normal volemia and plasma aldosterone levels (7). This raises the possibility that the site and mechanism of sodium retention in most nephrotic patients might be different from those described in PAN rats. As a matter of fact, when their plasma level of corticosteroids is clamped to basal level, PAN rats still develop nephrotic syndrome and amiloride-sensitive edema and ascites, but the mechanism of sodium retention is different because they display no membrane expression and activity of ENaC in the ASDN (6, 8). The principal aims of this study were to identify the amiloride-sensitive and ENaC-independent sodium reabsorption pathway responsible for sodium retention in these animals and its mechanism of induction. The secondary aim was to evaluate whether this alternate sodium reabsorption pathway is present in nephrotic patients.

Results show that sodium retention in corticosteroid-clamped (CC) PAN (CC-PAN) rats originates from ASDN and is dependent on the expression of a short variant of the acid-sensing ion channel (ASIC) 2b (ASIC2b) regulatory subunit that confers to the transiently active ASIC2a the properties of a long-lasting ENaC. Expression of this ASIC2b variant during nephrotic syndrome is secondary to albumin endocytosis and activation of ERK pathway in the ASDN. Expression of ASIC2 (mRNA and protein) was also found in ASDN from a majority of nephrotic patients.

## Results

### Sodium retention originated from collecting ducts of CC-PAN rats but was independent of the ENaC.

**Figure 1:**
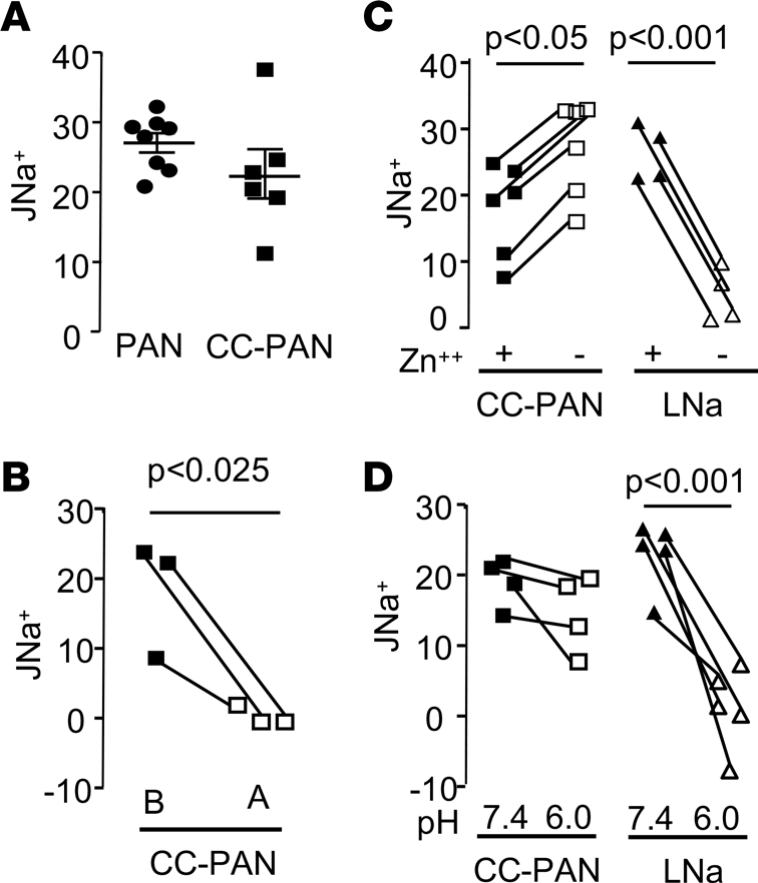
Sodium flux (J_Na_+) measured in microperfused CCDs in vitro. (**A**) J_Na_+ in CCDs from PAN nephrotic and CC-PAN nephrotic rats (PAN *n* = 8 and CC-PAN *n* = 6). Data are shown as mean ± SEM (each value represents a rat). (**B**) J_Na_+ was measured in CCDs from CC-PAN nephrotic rats before (**B**) and after (**A**) luminal addition of 10 μM amiloride. Each value represents a tubule (*n* = 3). (**C**) J_Na_+ in CCDs from CC-PAN rats and rats fed a LNa diet for 14 days before and after luminal addition of 300 μM ZnCl_2_. Each value represents a tubule (*n* = 6 and *n* = 4 in CC-PAN and LNa condition, respectively). (**D**) J_Na_+ in CCDs from CC-PAN and LNa rats was measured before and after acidification of the luminal fluid at pH 6.0. Each value represents a tubule (*n* = 4 and *n* = 5 in CC-PAN and LNa condition, respectively). Comparison between groups was performed by either 2-tailed unpaired *t* test (**A**) or paired *t* test (**B**–**D**). *P* < 0.05. CCDs, cortical-collecting ducts; CC, corticosteroid-clamped; LNa, sodium-depleted.

Measurement of the net transepithelial flux of sodium (J_Na_+) by in vitro microperfusion of isolated cortical-collecting ducts (CCDs) showed that in contrast to CCDs from control rats in which no J_Na_+ is measurable (9), CCDs from both PAN rats and CC-PAN rats displayed significant and similar J_Na_+ (Figure 1A). CCDs from PAN and CC-PAN rats also displayed a lumen^–^ transepithelial voltage (in mV ± SEM; PAN, –14.1 ± 2.2, *n* = 8; CC-PAN, –12.9 ± 1.9, *n* = 5; NS), indicating that sodium reabsorption was an electrogenic process. As previously reported in PAN rats (4), luminal addition of amiloride abolished J_Na_^+^ in CCDs from CC-PAN rats (Figure 1B) as well as the lumen^–^ transepithelial voltage (in mV ± SEM; control, –9.9 ± 4.7; amiloride, 5.9 ± 0.8, *n* = 3; *P* < 0.03). All these properties are consistent with an amiloride-sensitive process mediating sodium reabsorption in CCDs from CC-PAN rats. However, previous studies concluded to the absence of functional ENaC under these conditions (6, 8). Using sodium-depleted (LNa) rats as a model of overexpression of ENaC in the CCD (10), we searched for properties differentiating ENaC-mediated sodium transport from that in CC-PAN rats. J_Na_+ in CCDs from LNa rats was markedly reduced after luminal addition of 300 μM ZnCl_2_ whereas it was slightly increased in CC-PAN rats (Figure 1C). Luminal acidification (pH ≈ 6.0) abolished J_Na_+ in LNa rats but had no significant effect in CC-PAN rats (Figure 1D). Along with pieces of evidence previously reported (6, 8), these findings demonstrate that sodium retention in CC-PAN rats originates from the ASDN and stems from the activation of an electrogenic, amiloride-sensitive and Zn- and pH-insensitive transport pathway independent of ENaC.

### ASIC2 was responsible for J_Na_+ and sodium retention in CC-PAN rats.

**Figure 2:**
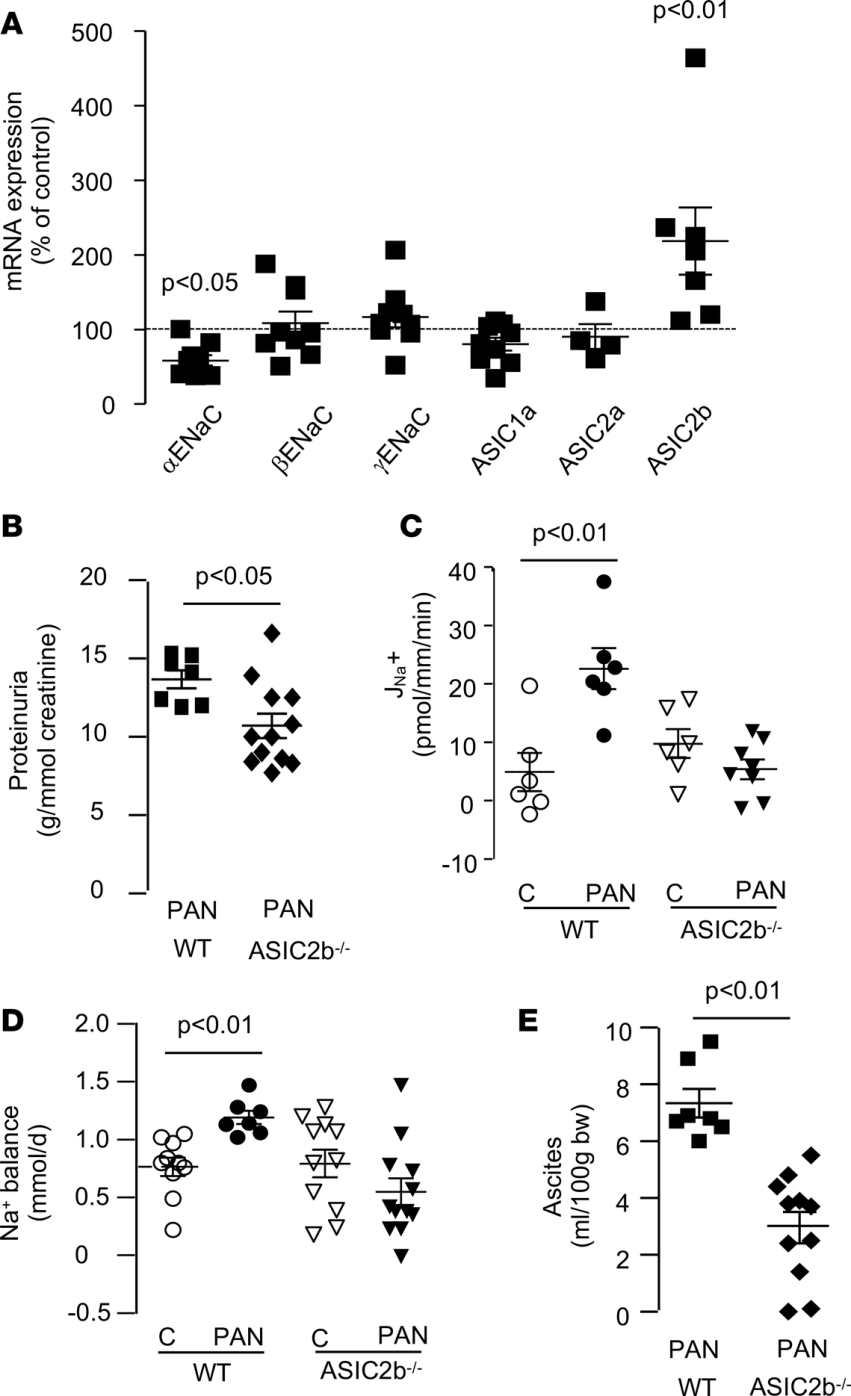
Role of ASIC2b in sodium retention in CC-PAN rats. (**A**) RT-qPCR analysis of ENaC/degenerin mRNA in CCDs from CC-PAN rats. Data are expressed as percent of values in CC control rats. Data are shown as mean ± SEM (each value represents a rat). (**B**) Proteinura in WT (*n* = 7) and ASIC2b^–/–^ CC-PAN (*n* = 13) rats. Proteinuria is expressed as a function of creatinine excretion. Data are shown as mean ± SEM (each value represents a rat). (**C**) J_Na_+ in CCDs from WT and ASIC2b^–/–^ rats under basal (**C**) or nephrotic (PAN) conditions. Data are shown as mean ± SEM (each value represents a rat, *n* = 6–7). (**D**) Urinary sodium balance in WT and ASIC2b^–/–^ rats under basal (**C**) or nephrotic (PAN) conditions. Data are shown as mean ± SEM (each value represents a rat, *n* = 7–12). (**E**) Volume of ascites, as a function of body weight, in WT (*n* = 7) and ASIC2b^–/–^ CC-PAN (*n* = 11) rats. Data are shown as mean ± SEM. Comparison between groups was performed by variance analysis (1-way ANOVA) followed by post hoc multiple comparison Tukey’s test (**A**) or by 2-tailed unpaired *t* test (**B**–**E**). *P* < 0.05. ASIC2b, acid-sensing ion channel 2b; CCDs, cortical-collecting ducts; CC, corticosteroid-clamped; ENaC, epithelial sodium channel.

We therefore looked for the expression of amiloride-sensitive channels of the ENaC/degenerin family in the CCD of CC-PAN rats, which includes the subunits of ENaC and ASICs (11). Like ENaC, which is a trimer of α, β, and γ subunits, ASICs are homotrimers or heterotrimers of ASIC1-5 channel isoforms. Beside the 3 subunits of ENaC, RT-qPCR revealed the mRNA expression of *Asic1a*, *Asic2a*, and *Asic2b*, whereas *Asic1b* and *Asic3– Asic5* were not detected. ASIC1a and ASIC2a can constitute functional channels by themselves, whereas ASIC2b cannot and is therefore considered as a regulatory subunit that associates with conductive isoforms and modifies their properties (12). Compared with CC control rats, the levels of *βENaC*, γ*ENaC*, *Asic1a*, and *Asic2a* mRNAs were unchanged in CCDs from CC-PAN rats and that of *αENaC* was reduced by 40%. In contrast, the expression level of *Asic2b* mRNA was increased 2-fold in the CCD from CC-PAN rat (Figure 2A). This suggests that ASIC2b in association with ASIC1a and/or ASIC2a may participate in J_Na_+ in CCDs and in sodium retention in CC-PAN rats. This hypothesis was confirmed by the finding that the genetic deletion of ASIC2b (see Supplemental Figure 1) abolished the stimulation of J_Na_+ in CCDs and sodium retention in CC-PAN rats and was reduced by over half the volume of ascites without altering proteinuria (Figure 2, B–E). The residual volume of ascites observed in CC-PAN ASIC2b^–/–^ rats is likely accounted for by the increased permeability of peritoneal capillaries described in PAN rats (13).

### Molecular characterization of a variant of ASIC2b in CC-PAN rats.

**Figure 3:**
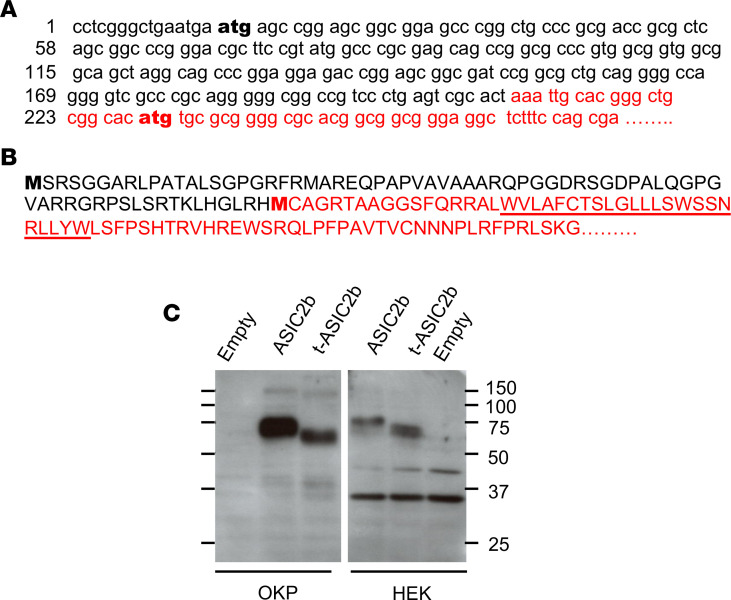
Long and truncated variants of ASIC2b. (**A**) 5′ terminal sequence of rat *Asic2b* cDNA (Accn1, transcript variant MDEG2, NM_012892, black) and of the short cDNA cloned from CC-PAN CCD (red, GenBank KP294334). The short sequence contains a putative translation initiation codon in frame with the ASIC2 ATG (in bold). (**B**) N-ter sequence of rat ASIC2b (black) and its putative truncated variant (red). The underlined sequence shows the first transmembrane domain. (**C**) Western blot analysis of ASIC2 expression in OKP and HEK cells transiently transfected with *Asic2b* or its truncated variant (*t-Asic2b*) or an empty vector. ASIC2b, acid-sensing ion channel 2b; CCDs, cortical-collecting ducts; CC, corticosteroid-clamped.

ASICs are mainly expressed in the nervous system, where their activation by extracellular acidification induces very brief cation currents that depolarize the cell membrane, allowing activation of nearby voltage-dependent channels or release of neuromediators (11, 14, 15). Given the transiency of ASIC-driven currents, ASICs cannot sustain epithelial sodium reabsorption. Therefore, we searched for a variant of ASIC2b that could convert ASIC1a or ASIC2a into a channel carrying sustained sodium current. Starting from RNAs extracted from a CC-PAN rat kidney, using 5′-RACE we generated a cDNA, the sequence of which was 100% identical to the deposited rat *Asic2b* sequence (*Accn1*, variant 1; NM_012892), except that it lacked the 207 5′-most nucleotides (Figure 3A). Interestingly, this truncated sequence (GenBank deposition KP294334) contains an ATG triplet (starting in position 22) within the same reading frame as the translation initiating codon of the full-length sequence, suggesting that this short sequence might be translated into a truncated variant of ASIC2b (t-ASIC2b) lacking the first 71 N-terminal amino acid residues, i.e., most of the N-terminal intracellular domain of ASIC2b (Figure 3B). The heterologous expression of the corresponding short cRNA in either OKP or HEK cells demonstrated that it is indeed translated into a protein of reduced molecular weight (Figure 3C).

**Figure 4:**
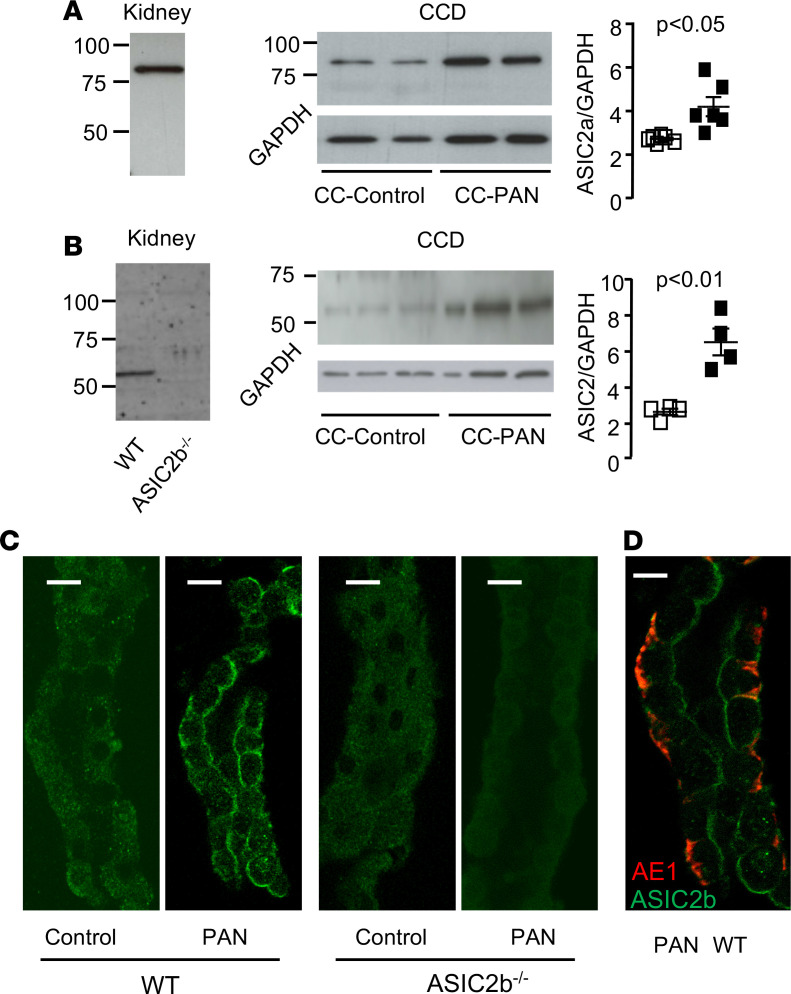
Renal expression of ASIC2. (**A** and **B**) Western blot analysis of ASIC2 expression in kidney of WT and ASIC2b^–/–^ CC-PAN rats and in CCDs of CC-Control and CC-PAN rats using a specific ASIC2a antibody (**A**) or a pan-ASIC2 antibody (**B**). Left, representative blot; right, densitometric analysis. Each value represents a rat. (**C**) Immunolabeling of isolated CCD from WT and ASIC2b^–/–^ CC-Control and CC-PAN rats with a pan-ASIC2 antibody. Scale bar: 10 μm. (**D**) Immunolabelling of isolated CCD from PAN WT rat with a pan-ASIC2 antibody (green) and an anti-AE1 antibody (red). Comparison between groups was performed by 2-tailed unpaired *t* test. *P* < 0.05. ASIC2, acid-sensing ion channel; CCDs, cortical-collecting ducts; CC, corticosteroid-clamped.

We next looked for the expression of t-ASIC2b and of ASIC1a and ASIC2a in the CCDs of CC-PAN rats. Immunoblotting with a specific anti-ASIC1a antibody (anti-ACCN2, sab2104215, MilliporeSigma) failed to reveal the expression of ASIC1a in the kidney of CC-PAN rats (data not shown). ASIC2a and ASIC2b are produced by alternative splicing of the same *Accn1* gene; they differ by their N-terminal domain consisting of 185 isoform–specific and 236 isoform–specific amino acid residues for rat ASCI2a and ASIC2b, respectively. An anti-ASIC2a–specific antibody (anti-ASIC2a, ASC-012, Alomone) revealed a single band approximately 80 kDa in kidney from CC-PAN rats. The same band was found in CCDs and its intensity was increased in CC-PAN rats compared with CC controls (Figure 4A), suggesting an overexpression of Asic2a, although its mRNA level was unchanged. An ASIC2b-specific antibody directed against an epitope present in full-length ASIC2b but not in t-ASIC2b (anti-rat MDEG2, MDEG21-a, Interchim) revealed no immunoreactivity in CC-PAN rat kidneys (data not shown), suggesting that the full-length ASIC2b protein was not expressed in the kidney of CC-PAN rats. In the absence of commercially available antibody specific for the t-ASIC2b protein, we used a pan anti-ASIC2 antibody directed against an epitope common to ASIC2a and ASIC2b (anti-ACCN1, ab77384, Abcam). This antibody revealed a faint band approximately 80 kDa (where ASIC2a was detected with the specific antibody) and a main band approximately 55 kDa, which was absent in the kidney of ASIC2b^–/–^ rats, indicating the presence of a t-ASIC2b protein in CC-PAN rat kidneys. The intensity of this band was higher in CCDs from CC-PAN rats compared with CC controls (Figure 4B).

Immunohistological labeling of isolated CCD with the pan-ASIC2 antibody demonstrated luminal expression of ASIC2a/b in CC-PAN rats but not in CC control rats, CC control ASIC2b^–/–^, or CC-PAN ASIC2b^–/–^ rats (Figure 4C). ASIC2 labeling was detected in both AE1^+^ cells (type A intercalated cells) and AE1^–^ cells (principal cells), as shown in Figure 4D.

### Functional characterization of t-ASIC2b/ASIC2a channels in Xenopus laevis oocyte.

**Figure 5:**
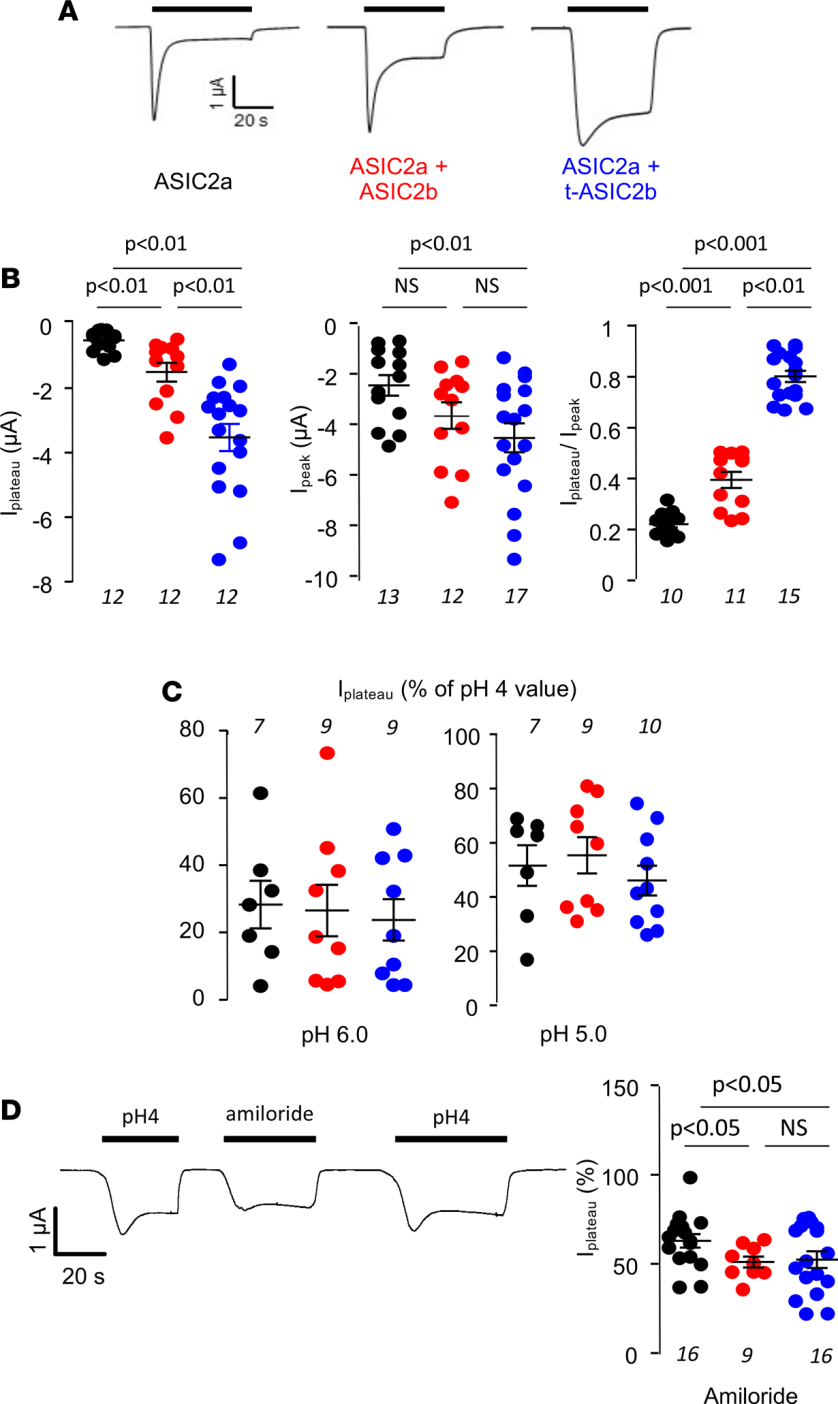
Functional expression of ASIC2 in *X. laevis* oocyte. (**A**) ASIC2b coexpression attenuates ASIC2a desensitization. Original traces of whole-cell current (holding potential = –70 mV) in oocytes expressing ASIC2a alone or with ASIC2b, or t-ASIC2b, as indicated below the traces. Inward currents were induced by a rapid extracellular acidification from pH 7.4 to pH 4.0 (indicated by the horizontal bar). (**B**) Mean transient-induced (peak) and residual-induced (plateau) currents (corrected by currents recorded in control oocytes) in oocytes expressing ASIC2a alone (black bars) or coexpressing ASIC2b (red) or t-ASIC2b (blue). (**C**) Acid-induced plateau currents (normalized to maximal value achieved at pH 4) at different extracellular pH; colors as above. (**D**) Effect of amiloride: acid-induced (pH 4) plateau currents (normalized to the value measured in the absence of inhibitor) in the presence of 100 μM amiloride. Data are shown as mean ± SEM (each value represents an oocyte, *n* is shown in italic in each figure). Comparison between groups was performed by variance analysis (1-way ANOVA) followed by post hoc multiple comparison Tukey’s test. *P* < 0.05. ASIC2, acid-sensing ion channel; t-ASIC2b, truncated variant of ASIC2b.

We next evaluated whether t-ASIC2b is functional and may alter ASIC2a properties by expression in *X*. *laevis* oocytes. Two-electrode voltage clamp experiments showed that expressing t-ASIC2b alone did not induce any acid-sensitive ion current in oocytes (data not shown), whereas its coexpression with ASIC2a modified the acid-induced current mediated by the latter. Figure 5A shows representative traces obtained at a holding potential of –70 mV in oocytes expressing ASIC2a alone or combinations of ASIC2a with either ASIC2b or t-ASIC2b. As previously described (16), decreasing the extracellular pH from 7.4 to 4.0 rapidly induced a transient current that spontaneously inactivated almost completely within seconds in ASIC2a-expressing oocytes. The coexpression of ASIC2a with the full-length ASIC2b increased the initial (peak) current and decreased the level of inactivation, leading to an enlarged residual (plateau) current. These changes were markedly amplified by the coexpression of t-ASIC2b with ASIC2a. The ratio of the plateau over the peak currents was significantly higher in oocytes coexpressing ASIC2a and t-ASIC2b compared with those expressing ASIC2a alone or in combination with ASIC2b (Figure 5B). In oocytes coexpressing ASIC2a and t-ASIC2b, the residual desensitized current remained stable for more than 5 minutes (data not shown). These findings indicate that the coexpression of ASIC2a with a N-ter truncated form of ASIC2b induced the formation of functional channels that allowed sustained transport of sodium in response to an acid stimulus. In the following experiments, we focused our analysis on the residual current carried by the desensitized channel, the one which can account for sodium reabsorption in CCDs.

Within the range of pH tested (7.4 to 4.0), the pH sensitivity of the plateau current was similar in oocytes injected with ASIC2a alone or in combination with ASIC2b or t-ASIC2b (Figure 5C). Truncated ASIC2b did not alter significantly the apparent low sensitivity of ASIC2a to amiloride (Figure 5D). The coexpression of t-ASIC2b changed neither the sodium affinity nor the cation selectivity of ASIC2a (data not shown).

### Putative posttranslational modifications of t-ASIC2b.

**Figure 6:**
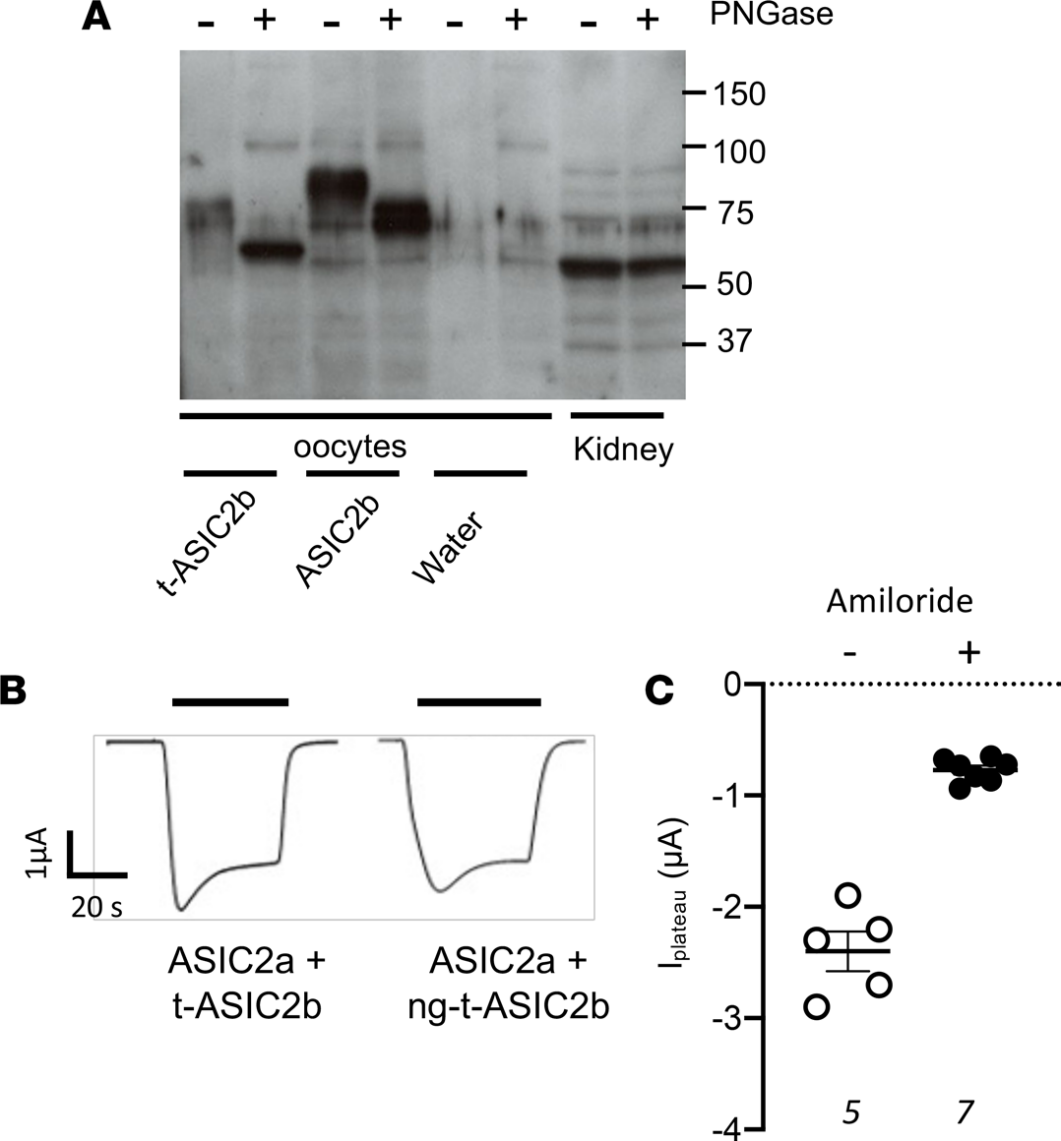
Glycosylation of t-ASIC2b. (**A**) Western blot analysis of ASIC2 expression in *X*. *laevis* oocytes injected with ASIC2b or t-ASIC2b cRNA or water and in protein extracts from CC-PAN rat kidneys. Samples were treated or not with N-glycosidase F (PNGase). (**B**) Original traces of whole-cell current (holding potential = –70 mV) in oocytes expressing ASIC2a with t-ASIC2b or a nonglycosylable form of truncated ASIC2b (ng-t-ASIC2b). Inward currents were induced by a rapid extracellular acidification from pH 7.4 to pH 4.0 (indicated by the horizontal bar). (**C**) Acid-induced (pH 4) plateau currents in the absence or presence of 100 μM amiloride. Data are shown as mean ± SEM (each value represents an oocyte, *n* is shown in italic in the figure). Comparison between groups was performed by 2-tailed unpaired *t* test. P < 0.05. t-ASIC2b, truncated variant of ASIC2b; ASIC2b, acid-sensing ion channel 2b; t-ASIC2b, truncated variant of ASIC2b; CC, corticosteroid-clamped.

The comparison of the apparent molecular weight of native t-ASIC2b (around 55–60 kDa; Figure 4B) and that of the transfected one in OKP or HEK cells (around 65 kDa; Figure 3B) suggests a putative, specific posttranslational modification in the kidney. To try to explain this discrepancy, we showed that the native t-ASIC2b was not sensitive to PNGase, whereas the t-ASIC2b expressed in oocyte was glycosylated (Figure 6A). The absence of glycosylation, obtained after directed mutagenesis of the 2 glycosylation sites (N416 and N443), did not modify the function of t-ASIC2b when expressed in oocytes, giving a similar amiloride-sensitive current as the WT t-ASIC2b (Figure 6, B and C).

### Expression of ASIC2a/b in nephrotic patients.

**Figure 7:**
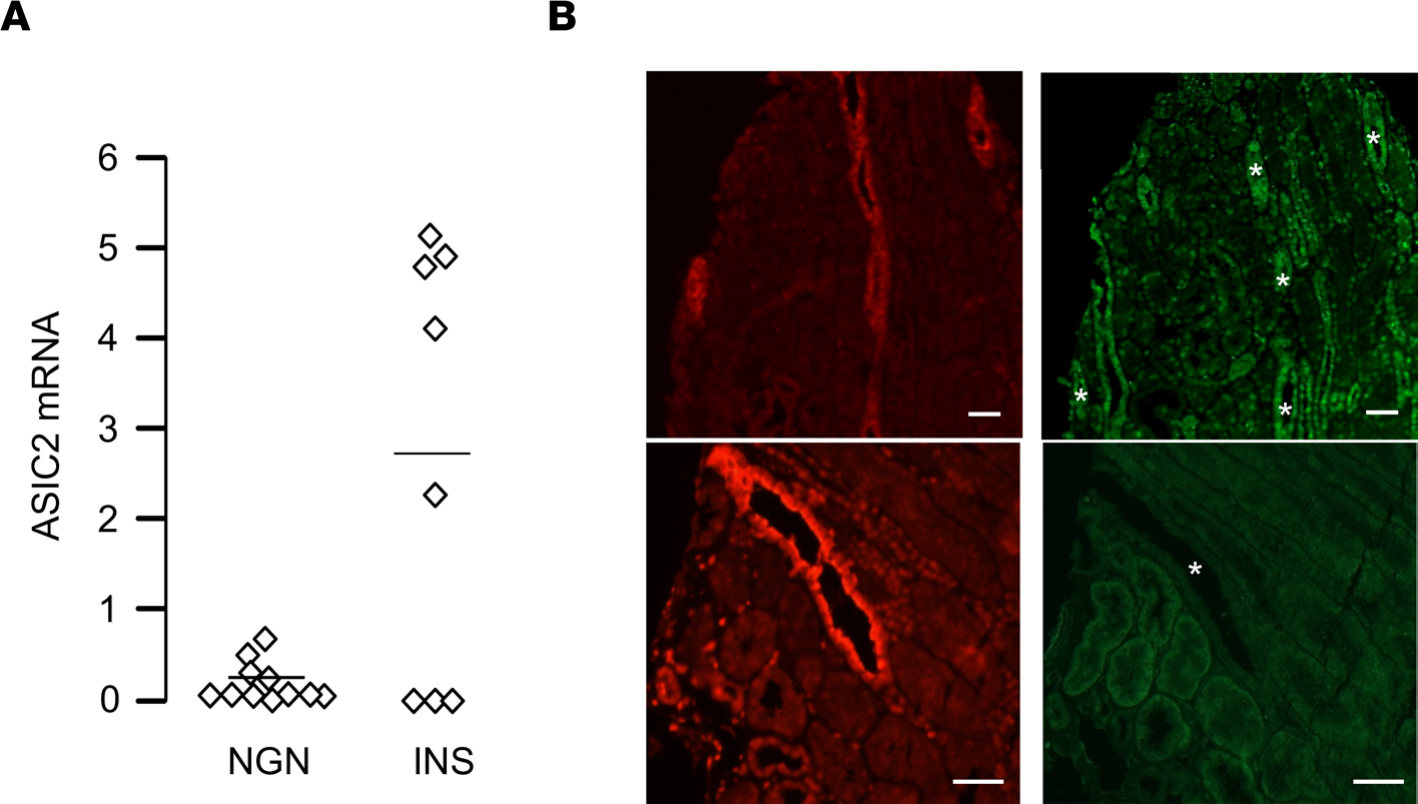
Expression of ASIC2 in nephrotic patients. (**A**) RT-qPCR analysis of *ASIC2* mRNA in kidney biopsies from patients with NGN (*n* = 11) or INS (*n* = 8). Given the heterogeneity of biopsies in term of cell composition, data were standardized using distal nephron markers, as previously described (38). Data are in arbitrary units. (**B**) Immunolabeling of kidney serial sections with anti-AQP2 (red) and anti-ASIC2 antibody (b and d, green) from a patient with INS (upper panels) or a nonnephrotic patient (lower panels). Scale bar: 100 μm. Comparison between groups was performed by 2-tailed unpaired *t* test. *P* < 0.05. ASIC2, acid-sensing ion channel; NGN, nonglomerular nephropathies; INS, idiopathic nephrotic syndrome.

We next evaluated whether ASIC2a/b is also expressed in the kidney of patients with minimal change disease. RT-qPCR revealed the presence of *ASIC2a/b* mRNA in kidney biopsies from 5 of 8 patients with INS, whereas it was undetectable in the remaining 3 patients (Figure 7A). All patients with nonglomerular renal diseases (11 of 11) displayed no expression of *ASIC2a/b* mRNA or a very low expression of *ASIC2a/b* mRNA. IHC showed ASIC2a/b labeling in collecting ducts of some, but not all, nephrotic patients, whereas labeling was never observed in nonnephrotic patients (Figure 7B).

### Signaling of ASIC2b induction during nephrotic syndrome.

**Figure 8:**
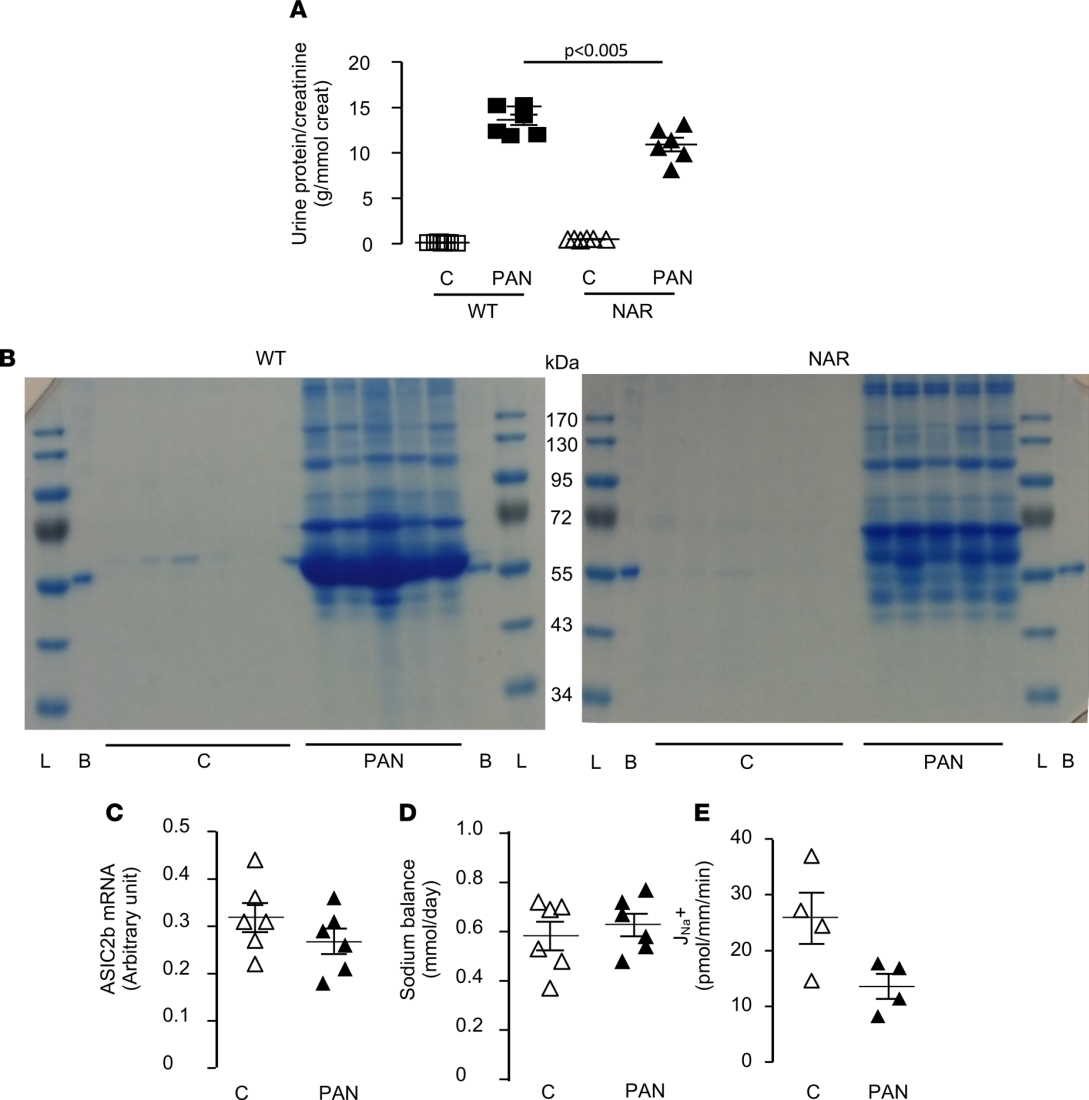
Role of albumin in ASIC2b expression and sodium retention. (**A**) PAN-induced proteinuria in CC-WT rats (*n* = 6) and NARs (*n* = 6). Proteinuria is expressed as a function of creatinine excretion. Data are shown as mean ± SEM. (**B**) Urinary proteinogram in WT rats and NAR under control (**C**) and nephrotic conditions (PAN). L, molecular weight markers; (**B**), BSA. (**C**) Expression of *Asic2b* mRNA in control (*n* = 6) and PAN nephrotic (*n* = 6) CC-NARs. Data are shown as mean ± SEM. (**D**) Sodium balance in control (*n* = 6) and PAN nephrotic (*n* = 6) CC-NARs. Data are shown as mean ± SEM. (**E**) J_Na_+ in CCDs from control (*n* = 4) and PAN nephrotic (*n* = 4) CC-NARs. Data are shown as mean ± SEM. Comparison between groups was performed by variance analysis (1-way ANOVA) followed by post hoc multiple comparison Tukey’s test (**A**) or by 2-tailed unpaired *t* test (**C**–**E**). *P* < 0.05. ASIC2b, acid-sensing ion channel 2b; CC, corticosteroid-clamped; NARs, Nagase analbuminemic rats; CCDs, cortical-collecting ducts.

It has been reported that albumin abnormally present in the kidney ultrafiltrate of nephrotic rats is endocytosed in CCDs, and that this process triggers several intracellular signaling cascades (17, 18). We therefore investigated the role of albumin in the induction of ASIC2b in CC-PAN rats. For this purpose, we used Nagase analbuminemic rats (NARs), a strain spontaneously lacking the albumin gene. Despite analbuminemia, CC-NARs developed massive but slightly lower proteinuria than control rats in response to PAN (Figure 8A). This proteinuria mainly consisted of proteins of higher molecular weight than albumin (Figure 8B). In CC-NARs, PAN increased neither *Asic2b* mRNA expression in CCD nor sodium retention (Figure 8, C and D). The volume of ascites was reduced by half in NARs compared with WT rats (in mL/100 g body wt ± SEM; WT, 7.3 ± 0.5, *n* = 6; NAR, 3.8 ± 0.5, *n* = 6, *P* < 0.001). Interestingly, J_Na_+ in CCDs from nephrotic NARs was not increased compared with nonnephrotic NARs (Figure 8E), indicating that albumin participated in the stimulation of ASIC2-dependent Na^+^ reabsorption.

**Figure 9:**
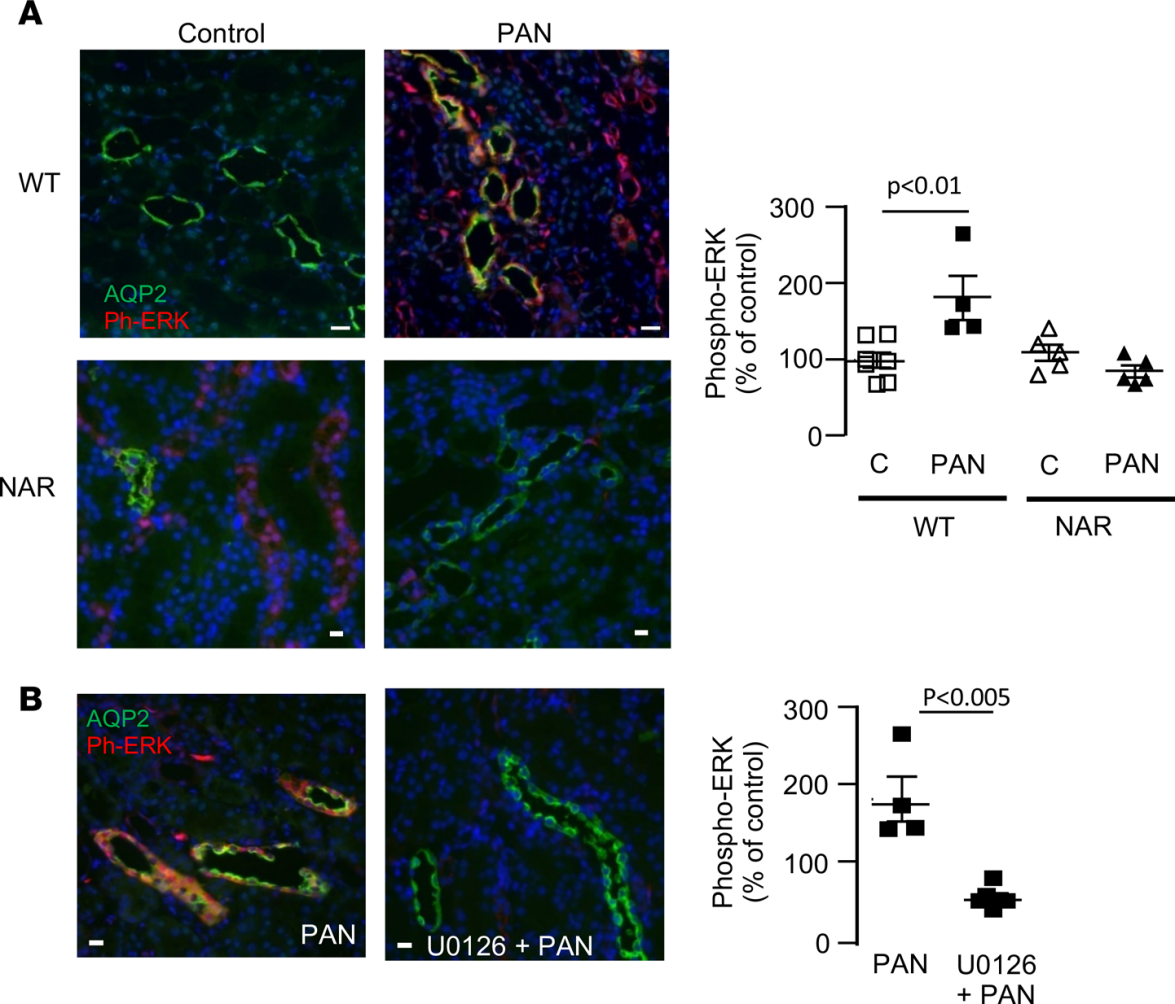
ERK pathway is triggered by luminal albumin. (**A**) Immunolabeling of kidney cortical sections from CC-WT rats and CC-NARs under control and PAN nephrotic condition with anti-phospho-ERK (red) and AQP2 (in green). AQP2 was used as a marker of CCDs. Left side shows representative images of phosphorylation of ERK in nephrotic WT rats but not in NARs. Right side shows the quantification of phospho-ERK labeling in CCDs. Values were normalized to the labeling measured in each experiment in the same CC control WT rat. Data are shown as mean ± SEM (*n* = 4–8). (**B**) Same immunolabeling as in **A** in CC-PAN WT rats treated or not with the ERK kinase inhibitor U0126. Data are shown as mean ± SEM (*n* = 4–6). Comparison between groups was performed by variance analysis (1-way ANOVA) followed by post hoc multiple comparison Tukey’s test (**A**) or by 2-tailed unpaired *t* test (**B**). *P* < 0.05. CCDs, cortical-collecting ducts; CC, corticosteroid-clamped.

**Figure 10:**
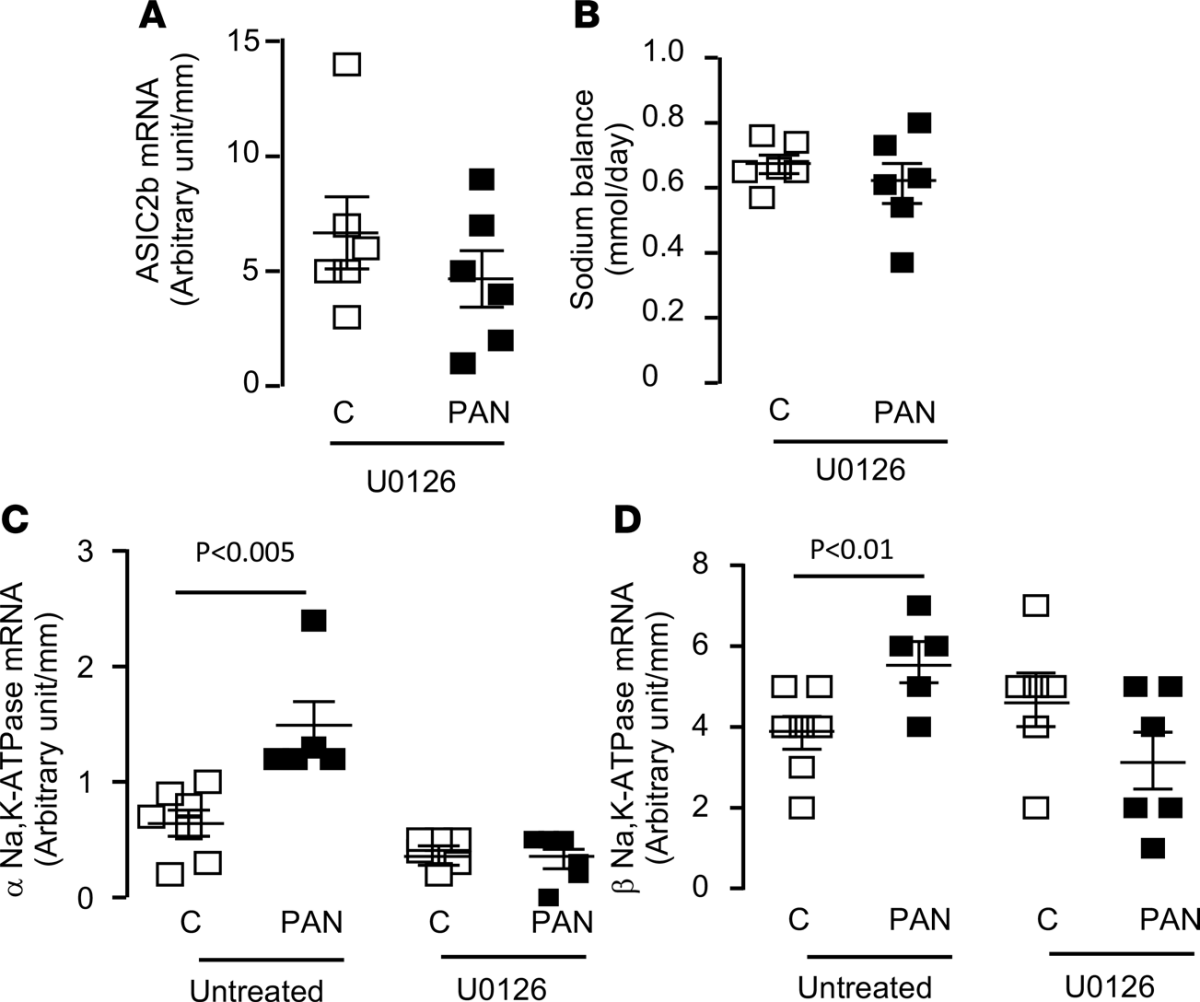
Role of ERK pathway in ASIC2b expression and sodium retention. (**A**) Expression of *Asic2b* mRNA in U016-treated rats under control (*n* = 6) and nephrotic conditions (*n* = 6). Data are shown as mean ± SEM. (**B**) Sodium balance in U016-treated rats under control (*n* = 6) and nephrotic conditions (*n* = 6). Data are shown as mean ± SEM. (**C** and **D**) Expression of mRNAs of the α and β subunits of Na,K-ATPase in U0126-untreated and U0126-treated rats under control and nephrotic conditions. Data are shown as mean ± SEM (*n* = 5–8). Comparison between groups was performed by 2-tailed unpaired *t* test. *P* < 0.05. ASIC2b, acid-sensing ion channel 2b.

We next evaluated the possible involvement of ERK pathway in the induction of ASIC2b because we previously reported that this pathway is activated by albuminuria (18). By semiquantitative immunofluorescence, we confirmed that phospho-ERK labeling was increased in CCDs from CC-PAN rats compared with CC rats, and that this effect was abolished in NARs (Figure 9A), indicating that ERK phosphorylation was induced by albumin, probably through its endocytosis. ERK phosphorylation was also prevented by in vivo treatment of CC-PAN rats with the mitogen-activated protein kinase inhibitor U0126 (Figure 9B). U0126 treatment also abolished the induction of *Asic2b* mRNA expression and the positivation of sodium balance (Figure 10, A and B), and over half the volume of ascites was reduced (in mL/100 g body wt ± SEM; control, 7.3 ± 0.5, *n* = 6; U0126, 3.0 ± 0.4, *n* = 5, *P* < 0.001). We also found that U0126 prevented the overexpression of the 2 subunits of Na,K-ATPase, the motor for sodium reabsorption in the CCD (Figure 10, C and D). Unfortunately, we were not able to dissect native CCDs from U0126-treated rats to measure J_Na_+ by in vitro microperfusion in these rats.

**Figure 11:**
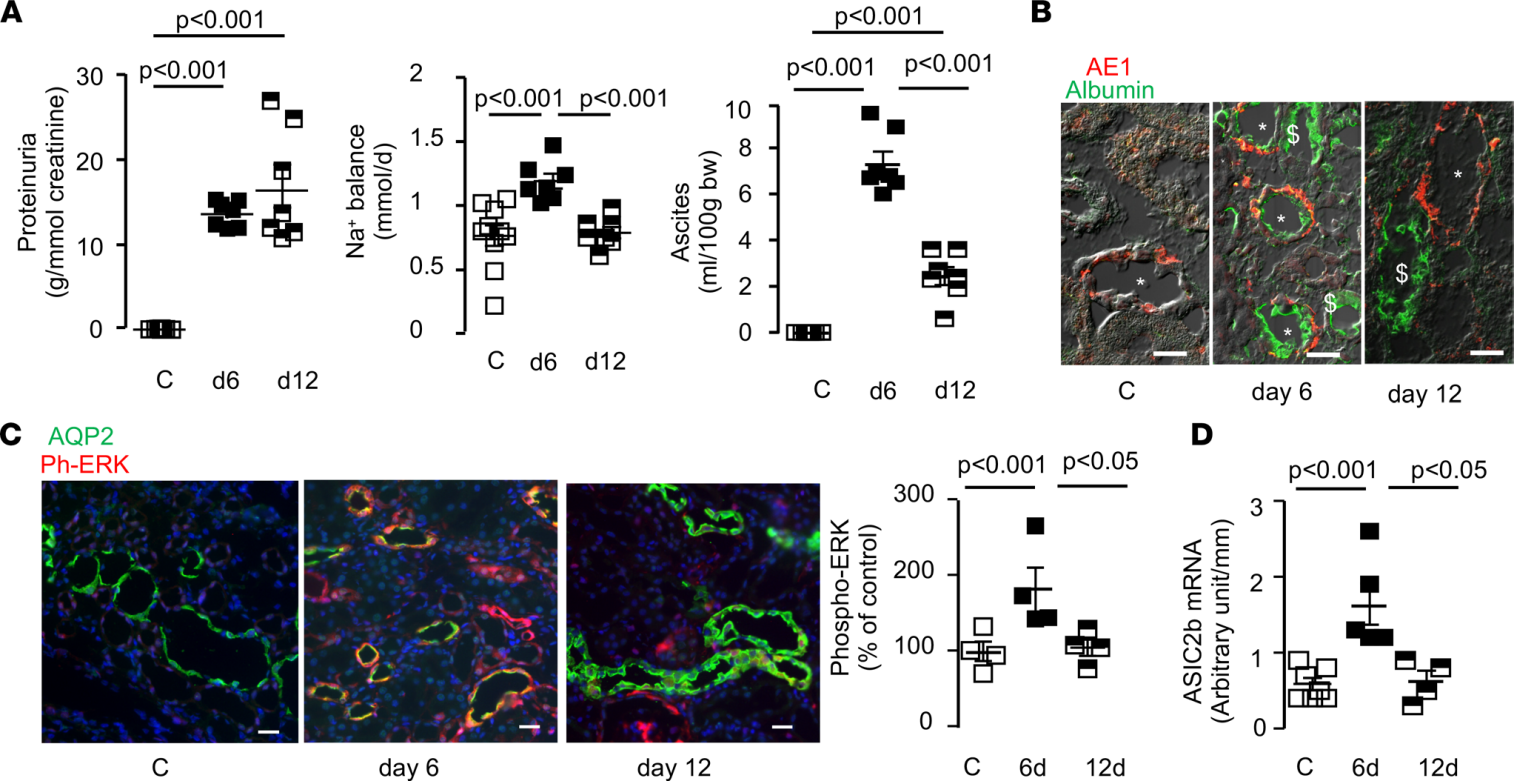
Reversal of sodium retention. (**A**) Proteinuria, sodium balance and volume of ascites in CC rats under control condition (**C**) or 6 or 12 days after PAN administration. Data are shown as mean ± SEM (*n* = 7–11). (**B**) Immunolabeling of kidney cortex section from CC rats under control condition (**C**) or 6 or 12 days after PAN administration with an anti-albumin antibody (green) and AE1 antibody (red), a specific marker of CCD intercalated cells. *, CCD; ^$^, proximal tubule. Scale bar: 25 μm. (**C**) Immunolabeling of kidney cortex section from CC rats under control condition (**C**) or 6 or 12 days after PAN administration with an anti-phospho-ERK antibody (red) and an anti-AQP2 antibody (green), a specific marker of CCD principal cells. Left, representative images; right, quantification as in Figure 8A. Data are shown as mean ± SEM (*n* = 4). (**D**) Expression of *Asic2b* mRNA in CCDs from CC rats under control condition (**C**) or 6 or 12 days after PAN administration. Data are shown as mean ± SEM (*n* = 4–7). Comparison between groups was performed by variance analysis (1-way ANOVA) followed by post hoc multiple comparison Tukey’s test. *P* < 0.05. CC, corticosteroid-clamped; CCDs, cortical-collecting ducts.

Altogether, these results indicate that nephrotic albuminuria activated the ERK pathway and subsequently induced the expression of ASIC2b and sodium retention in CCDs. This mechanism is specific for albumin. This conclusion raises a question regarding the reversal of sodium retention in PAN rats. As a matter of fact, it has been shown than within 12 days after the administration of PAN, sodium balance and Na,K-ATPase activity in CCD returned to basal levels and that ascites disappeared despite the maintenance of massive proteinuria. We confirmed that, within 12 days, the sodium balance was restored to basal level and ascites were reduced, whereas proteinuria remained high (Figure 11A). We observed that, at that time, albumin was no longer accumulated in the CCDs (Figure 11B) and that ERK phosphorylation and *Asic2b* mRNAs had turned back to basal levels (Figure 11, C and D).

## Discussion

Sodium reabsorption in the CCD proceeds via 2 pathways: the classical electrogenic pathway mediated by amiloride-sensitive ENaC on the apical side and basolateral Na,K-ATPase; and an electroneutral, thiazide-sensitive pathway energized by the basolateral H-ATPase and requiring the concerted activity of apical sodium-dependent and sodium-independent chloride/bicarbonate exchangers and a basolateral sodium bicarbonate cotransporter (10, 19). These 2 pathways originate from principal cells and type B intercalated cells, respectively. In contrast, type A intercalated can secrete sodium via basolateral Na/K/Cl cotransporter and apical H(Na),K-ATPase (20, 21). Here we show that the sodium reabsorption in CCDs from CC-PAN rats was electrogenic and amiloride-sensitive but independent of any ENaC subunit, because none of them was expressed at the apical cell border (6), revealing the existence of a third pathway for sodium reabsorption. This pathway involves a newly characterized truncated variant of ASIC2b, which, in association with ASIC2a, can carry sustained apical sodium entry. This channel was expressed at the apical border of all cell types constituting CCDs, suggesting that they might all have participated in sodium reabsorption. However, this appeared unlikely for type A intercalated cells because they did not express any known sodium pump at their basolateral side.

Based on coimmunoprecipitation and functional studies, Ugawa and coworkers have shown that rat ASIC2a and ASIC2b biochemically assemble to constitute functional (16) channels. The variant of ASIC2b identified in this study differs from ASIC2b by the deletion of most of its intracellular N-terminal domain (71 of 88 amino acid residues). The N-terminal domain of ASICs is not necessary for subunit association because the trimeric structure of chicken ASIC1 was deduced from crystal structures obtained from proteins with truncated intracellular domains (22, 23). This indicates that ASIC2a and truncated ASIC2b can assemble to constitute functional channels likely made of 3 subunits like all functional ASICs. However, the stoichiometry of assembly remains unknown and may be variable in vivo as well as in our coexpression studies. Consequently, the macroscopic currents measured in *X*. *laevis* oocytes may have stemmed from a mix of 3 types of trimeric channels containing 0, 1, or 2 subunits of t-ASIC2b.

Current kinetics models propose that ASICs exist under 3 functional states — a closed state, an open state, and a desensitized state, in which the channel is partially open but cannot be activated by acid stimulus. After acid stimulus, the open and desensitized states translate into peak and plateau currents, respectively. It was previously reported that the main effect of ASIC2b is to increase the current carried by desensitized ASIC2a (16). We confirmed this effect of ASIC2b and found that it is amplified by t-ASIC2b. Thus, the kinetics of the inward current mediated by ASIC2a/t-ASIC2b resembles that of ENaC-mediated current, except for the presence of a transient peak of small amplitude as regards to the remaining plateau. Another important issue concerns the intensity of the current carried by ASIC2a/t-ASIC2b compared with ENaC-mediated current. Formally, this question cannot be answered by electrophysiological analysis in *X*. *laevis* oocyte because the current intensity depends on the expression level of the respective channels and possibly on the stoichiometry of t-ASIC2b/ASIC2a assembly. Nonetheless, it is worth mentioning that the macroscopic Na^+^ current measured in ASIC2a and t-ASIC2b-expressing oocytes under the desensitized state (~3 μA at a holding potential of –70 mV) is of the same order of magnitude as that initially reported in ENaC-expressing oocytes (~1 μA at –100 mV; ref. 24). Altogether our findings indicate that given their conductive properties under desensitized state, heterotrimers made of ASIC2a and t-ASIC2b may be substituted for ENaC and may have allowed sustained sodium reabsorption in CCD principal cells.

Conversely to ENaC, which opens stochastically in the absence of stimulus, ASIC2 requires an acid stimulus to open and to desensitize. Thus, the increased abundance of ASIC2 subunits in CCDs from CC-PAN rats is not sufficient to account for sodium reabsorption; this also requires the presence of an ASIC2-activating factor. ASIC2a requires low pH for maximal activation (pH_50_ ~4.0, maximal current, pH ~2.0; ref. 16), and association with t-ASIC2b did not modify this pH dependency. The pH of the luminal fluid prevailing in CCDs in vivo is estimated in the 6.0–6.5 range (25, 26), but because type A intercalated cells of the CCD secrete protons and due to the presence of unstirred layers, the pH at the external surface of the apical membrane might be 0.5–1.0 units lower, i.e., in the 5.0–6.0 range. Based on *Xenopus* *laevis* expression experiments shown in Figure 5C, these pH levels induced 20%–40% of the current carried by desensitized ASIC2a/t-ASIC2b (I_plateau_) at pH 4.0, which may be sufficient to account for the rate of sodium reabsorption determined by in vitro microperfusion. In addition, one cannot exclude that the factors abnormally present in the urine of nephrotic rats or produced by CCD cells may also have increased the pH sensitivity of ASIC2a/t-ASIC2b or activated it independently of pH. Such mechanisms have been reported with other ASICs, e.g., NO and arachidonic acid potentiate ASIC-mediated proton-gated currents (27, 28), and the arginine metabolites agmatine and arcaine activate ASIC in a proton-independent manner (29, 30). Moderate proteolysis of ASIC2 may also activate it, as previously reported for ENaC and plasmin (31), the excretion of which is increased during nephrotic syndrome (32).

The apparent low amiloride sensitivity of t-ASIC2b/ASIC2a observed in *X*. *laevis* oocytes (IC_50_ ≈50 μM) contrasts with the high sensitivity of sodium transport observed in vitro in microperfused CCDs (full inhibition with 10 μM amiloride). These differences in amiloride sensitivity likely stem from the fact that acid pH required to activate ASIC2 in *X*. *laevis* oocytes strongly decreases its sensitivity to amiloride (16). This further supports the notion that acid pH is probably not the unique activating factor of ASIC2 in ASDN during nephrotic syndrome.

The mechanism of sodium retention in nephrotic rats varies according to their aldosterone status: when animals display high levels of plasma aldosterone, increased sodium reabsorption is mediated by the classical ENaC-dependent pathway and there is no evidence for ASIC2 expression, whereas blunting of hyperaldosteronemia switches off the ENaC pathway and triggers the ASIC2-dependent one. This suggests the existence of a balance between factors that reciprocally trigger and repress the renal expression of ENaC and ASIC2. Aldosterone is the major inducer of ENaC expression in the ASDN; whether or not it represses the expression of ASIC2 remains to be established. Here we report that the phosphorylation of ERK brought about by the endocytosis of albumin mediated the overexpression of ASIC2 in the ASDN and the stimulation of sodium transport. In contrast, ERK phosphorylation reduces ENaC activity by different mechanisms including the decrease of its open probability, increasing its membrane retrieval (33), and decreasing the expression of its mRNA expression (34). Thus, endocytosis of albumin and subsequent activation of ERK pathway appears as a major factor that switches on and off ASIC2 and ENaC pathways, respectively. This raises the question of the mechanism responsible for the escape of sodium retention to albuminuria observed in the long term. Our data show that it proceeds at the step of albumin endocytosis, possibly via the downregulation of the albumin receptor 24p3R (17).

Our preliminary results in nephrotic patients indicate that the expression of ASIC2 in the ASDN was not restricted to the PAN rat model. The fact that only portion of the patients studied displayed ASIC2 expression in their ASDN might be related to the fact that, in nephrotic rats, ASIC2 is found only in CC animals and only a fraction of nephrotic patients displays normal aldosterone status (7). A further study will be necessary to evaluate whether the expression of *Asic2* in nephrotic patients is correlated with low plasma aldosterone level. More generally, it is noteworthy that the alternate ATG-initiating codon found in the rat *Asic2b* sequence is conserved not only in the human sequence (NM_183377.1) but also in mice (NM_007384.3).

It has been previously reported that channels made of αENaC and ASIC1a constitute functional cation-reabsorbing channels that participate in fluid clearance by lungs (35). Here we describe the functional expression and role of channels exclusively made of ASIC subunits out of excitable cells. It also shows that the deletion of the intracellular N-terminal domain of ASIC2b modified its properties and converted the transient ASIC2a into a long-lasting ENaC. Thus, ASICs and ENaC shared more than sequence similarities because both performed epithelial sodium transport. Whether variants of ENaC might work as transient sodium channels is a stimulating hypothesis.

## Methods

### Animals.

Experiments were carried out on male rats (150–170 g at the onset of the experimentation) fed a standard laboratory chow (A04, SAFE) with free access to water. Sprague-Dawley rats were from Charles Rivers and NARs were from Japan SLC. For surgery, animals were anesthetized by i.p. injection of a mix including Domitor (0.5 μg/g body wt; Pfizer), Climasol (2 μg/g body wt; Graeub), and Fentanyl Janssen (5 ng/g body wt; Janssen Cilag Lab). Animals were awakened by a s.c. injection of a mix containing Antisedan (750 ng/g body wt; Pfizer), Sarmasol (200 ng/g body wt; Graeub), and Narcan (133 ng/g body wt; Aguettant). Before killing, animals were anesthetized (i.p.) with pentobarbital (50 mg/kg body wt; Sanofi). CC was achieved by bilateral adrenalectomy and supplementation with aldosterone (10 μg/kg/d) and dexamethasone (14 μg/kg/d) through s.c. osmotic pump (ALZET, Charles River; ref. 6). Nephrotic syndrome was induced the day after surgery for CC by a single intrajugular injection of aminonucleoside puromycine (PAN; 150 mg/kg body wt; MilliporeSigma). Control rats received a single injection of isotonic NaCl (1 mL/100 g body wt). A group of rats was treated with the ERK kinase inhibitor U0126 by daily s.c. injection (3 mg/10 g body wt/d in a mixture of DMSO/sesame oil, 16%/84% v/v). Treatment started the day of CC. Animals were studied 6 days after vehicle or PAN injection, at the time of maximum of sodium retention and proteinuria, or after 12 days when sodium balance was restored (36). To induce ENaC expression, rats were fed a Na^+^-depleted diet (synthetic diet containing 0.11 g Na^+^/kg instead of 2.5 g/kg; SAFE). Rats were studied 14 days after the onset of the low Na^+^ diet.

### Generation of ASIC2b-null rats.

Invalidation of *Accn1* gene encoding the ASIC2 proteins was performed using CRISPR/Cas9 technology (TRIP, INSERM UMR1064). Zygotes from Sprague-Dawley rats were microinjected with a single-guide RNA (10 ng/μL) designed to target exon 1 of the *Accn1* gene and Cas9 mRNA (50 ng/μL) as previously described (37). This resulted in a deletion of 20 nt and a frameshift of the coding region leading to the early appearance of a premature stop codon. Embryos were then implanted in pseudopregnant females and grown until full term. To genotype the animals, a 953 pb genomic DNA fragment around the targeted region was PCR to detect the deleted fragment in KO animals by gel electrophoresis.

### Urine proteinogram.

Urine samples were thawed and centrifuged 10 minutes at 3300*g*. Supernatant (9 μL; pure for control rats, diluted 1:10 in H_2_O for PAN rats) were half diluted in glycerol/blue solution and separated by SDS-PAGE. After migration gels were rinsed twice with water and stained overnight in Coomassie blue solution (PageBlue Protein Staining, Thermo Fisher Scientific) at room temperature. The gels were then rinsed for at least 48 hours in water.

### Microdissection of CCDs.

CCDs were dissected under stereomicroscopic observation either from fresh kidney slices (microperfusion) or after liberase treatment (Blendzyme 2, Roche Diagnostics; immunoblotting, RT-qPCR, and IHC; ref. 18). For RT-qPCR experiments, microdissection was performed under RNase-free conditions.

### In vitro microperfusion.

Unless indicated otherwise, CCDs were perfused at 37°C under symmetrical conditions (7), with bath and perfusate containing (in mM): 138 NaCl, 1.2 MgSO_4_, 2 K_2_HPO_4_, 2 calcium lactate, 1 sodium citrate, 5.5 glucose, 5 alanine, and 12 creatinine (bath continuously gassed with 95% O_2_/5% CO_2_). pH was adjusted to either 7.4 with Hepes 10 mM and Tris 5 mM or 6.0 with MES 5 mM. Transepithelial voltage (PD_te_) was measured via microelectrodes connected through an Ag/AgCl half-cell to an electrometer. Tubules were perfused at a low rate (~2 nL/min). Concentrations of Na^+^, and creatinine were determined by HPLC, and ion flux (JNa^+^) was calculated as: J_Na+_ = [([Na^+^]_p_ × V_p_) — ([Na^+^]_c_ × V_c_)] L × t, where [Na^+^]_p_ and Na^+^]_c_ are the concentration of Na^+^ in the perfusate and collection, respectively, V_p_ and V_c_ are the perfusion and collection rates, respectively, L is the tubule length, and t is the collection time. For each tubule, Na^+^ flux was calculated as the mean of 4 successive 10–15–minutes collection periods. V_p_ was calculated as: *V*_p_ = *V*_c_ × [creat]_c_*/*[creat]_p_ where [creat]_c_ and [creat]_p_ are the concentrations of creatinine in the collection and perfusate, respectively.

### RNA extraction and RT-qPCR.

RNAs were extracted from 40–50 CCDs using the RNeasy Micro Kit (QIAGEN) as previously described (18). Frozen human kidney biopsies (2–3 mg) were dissolved in 5 μL of RLT complemented with β-mercaptoethanol and transferred in a tube, and RNAs were extracted as above. RNAs were reverse-transcribed using First Strand cDNA Synthesis Kit for RT-PCR (Roche Diagnostics) according to the manufacturer’s protocols. RT-PCR was performed with a LightCycler 480 SYBR Green I Master Quantitative PCR Kit (Roche Diagnostics) according to the manufacturer’s protocol, except that the reaction volume was reduced 2.5-fold. Specific primers (Table 1) were designed using Probe Design (Roche Diagnostics).

In each experiment, a standardization curve was made using serial dilutions of a standard cDNA stock solution made from rat or human kidney, and data (in arbitrary units) were calculated as function of the standard curve. For CCDs, results were normalized as a function of Rps23 expression. Data are mean ± SEM from several animals. Given the heterogeneity of human kidney biopsies in terms of cell composition, data were normalized using the distal nephron markers AQP2 and FXYD4, as previously described (38).

### 5′-RACE.

Total RNA was extracted from the kidney of a CC-PAN rat following the Tri Reagent protocol (MilliporeSigma), DNAse I–treated, and purified using RNeasy Mini Kit (QIAGEN) according the manufacturer’s instructions. The 5′-RACE cDNA was synthesized by using the GeneRacer Kit (Invitrogen) according the manufacturer’s instructions. The first PCR reaction was performed using *Asic2b*-specific reverse primer (5′- cacaaggagtgtgcagagcctgc-3′) and the GeneRacer 5′ primer supplied with the RACE cDNA kit. The nested *Asic2b*-specific reverse primer (5′-cctttcatccaagagctgggctttg-3′) and the GeneRacer 5′ Nested primer supplied with the kit were used for the second PCR reaction. The nested PCR products were subcloned in the pCR4-TOPO vector (TOPO TA Cloning Kit for Sequencing, Invitrogen) according the manufacturer’s instructions and sequenced.

### ASIC2 subcloning.

The full-length rat *Asic2b* (*Accn1*, variant 1, NM_012892) was purchased from ImaGenes and cloned into pMK (kanR) using SfiI cloning sites. Asic2b N416A and Asic2b N443A mutants were generated using QuikChange Site-Directed Mutagenesis Kit (Agilent Technologies) according to the manufacturer’s instructions. For expression in *X*. *laevis* oocytes, the fragment of interest was extracted and subcloned in pGH19 expression vector Bam HI/Xba I cut. For expression in eukaryotic cells, the pcDNA 3.1/V5-His TOPO TA Expression Kit (Invitrogen) was used according to the manufacturer’s protocol. The Bam HI and Xba I sites were used to subclone the insert of interest.

The truncated variant of rat *Asic2b* was obtained as described above and subcloned in EcoRI site into pTLB expression vector for expression in *X*. *laevis* oocytes. Subcloning for expression in eukaryotic cells was performed as for full-length *Asic2b*.

A nonglycosylable mutant of t-Asic2b was generated with QuikChange Mutagenesis Kit (Agilent Technologies) following the manufacturer’s instructions. The 2 N-glycosylated sites (asparagine at positions 416 and 443 in the WT protein) were changed to alanine residues.

Full-length rat *Asic2a* (*Accn1*, variant 2, NM_001034014) was provided by E. Lingueglia (Institut of Molecular and Cellular Pharmacology [IMPC], Valbonne, France) in a mammalian vector and cloned in pGH19 *X*. *laevis* expression vector. All constructs were verified by sequencing.

### Expression in mammalian cells.

OKP and HEK293 cells were cultured in DMEM medium, high glucose, and glutamax (GIBCO, Thermo Fisher Scientific) and DMEM medium, high glucose, and Hepes, respectively, and supplemented was with 100 mM pyruvate. Both media were supplemented with 10% FBS (Invitrogen), penicillin (100 units/mL), and streptomycin (100 μg/mL). Cultures were incubated at 37°C in a humidified 5% CO_2_ air atmosphere. Cells grown for 24 hours in 6-well plates were transfected with 1 μg of cDNA construct (*Asic2b* or *t-Asic2b*) using a Lipofectamine Plus Kit (Invitrogen) according to the manufacturer’s protocol. Transiently transfected cells were studied 48 hours after transfection. Empty vector was used as control.

### Functional expression in X. laevis oocytes.

Linearized pGH19-*Asic* constructs were transcribed in vitro from the promoter using a mCAP mRNA Mapping Kit (mMESSAGE mMACHINE T7/SP6 Kit, Ambion).

Oocytes were obtained after the partial ovariectomy of female adult *X*. *laevis* (obtained from Xenopus Express, Vernassal, France). Stage V–VI oocytes were selected after enzymatic defolliculation (2 hours of gentle shaking in a collagenase-containing solution [1A, MilliporeSigma] at 16°C). Control oocytes were injected with 50 nL of RNase-free water. *Asic*-expressing oocytes were injected with 50 nL of RNase-free water containing 3 ng of *Asic2a* (oocytes expressing Asic2a alone) or a mixture of 3 ng of *Asic2a* cRNA plus 6 ng of *Asic2b* (oocytes coexpressing *Asic2a* and *A*sic2b). Oocytes were incubated for 2–4 days at 16°C before electrophysiological experiments or analysis of protein expression.

A 2-electrode voltage clamp was achieved using low resistance (<1 MΩ) glass microelectrodes, filled with 3 M KCl, and connected to a voltage-clamp amplifier (Turbo TEC-10CX, NPI Electronic). Whole-cell currents were recorded at the holding potential VH = –70 mV interfaced to a computer with Digidata 1322A and analyzed using Axon pClamp 9 software (Axon Instruments). The oocyte was continuously superfused during the experiment with ND 96 solution (in mM: NaCl 96, KCl 2, CaCl_2_ 1.8, MgCl_2_ 1, Hepes 5, pH 7.4) buffered with 5 mM HEPES and adjusted to pH 7.4 or with 5 mM MES and adjusted to pH 4–6 to elicit acid-induced currents.

### Immunoblotting.

Pools of 30–80 CCDs were solubilized in Laemmli buffer, and proteins were separated by SDS-PAGE and transferred to polyvinylidene difluoride membranes (GE Healthcare). After blocking, blots were successively incubated with specific anti-ASIC2a antibody (ASC-012, 1:1000, Alomone Labs; see Table 2) or pan-ASIC2 antibody (ab77384, 1:700, Abcam) and anti–rabbit IgG antibody coupled to horseradish peroxidase (W401B, 1:2500, Promega) and revealed with the Western Lightning Chemiluminescence Reagent Plus (PerkinElmer Life Sciences). After stripping, membranes were incubated with anti-GAPDH antibody (ab9485, 1:1000, Abcam) and posttreated as described above. Densitometry of the bands was quantitated by using NIH ImageJ software. Pools of 30 oocytes were solubilized in a buffer containing 5 mM Tris-HCl pH 7.4, 250 mM sucrose, 0.5 mM EDTA with cOmplete Protease Inhibitor Cocktail (Roche). For enzymatic deglycosylation, 15–20 μg of proteins were treated or not with peptide N-glycosidase F following the manufacturer’s protocols (New England BioLabs Inc.). Samples were mixed with Laemmli buffer and proteins were separated by SDS-PAGE and analyzed by Western blot using pan-ASIC2 antibody as described above for CCDs. Transfected cells with Asic2b constructs or empty vectors were solubilized in buffer containing 50 mM Tris-HCl pH 7.4, 150 mM NaCl, 2 mM EDTA, 1% (v/v) Triton X-100, 0.1% SDS, and cOmplete Protease Inhibitor Cocktail (Roche Diagnostics). Proteins were separated by SDS-PAGE and analyzed by Western blot using pan-ASIC2 antibody as described for CCDs.

### ASIC2 IHC.

Pools of 15–20 rat CCDs were transferred to Superfrost Gold (Thermo Fisher Scientific) glass slides, fixed with 4% paraformaldehyde, incubated for 20 minutes at room temperature in 100 mM glycine, and permeabilized for 30 s with 0.1% Triton. After blocking, slides were incubated with anti-pan-ASIC antibody (ab77384, 1:500, Abcam) for 1 hour at room temperature, and for 1 experiment, with an anti-AE1 (1:500; a gift from C. Wagner, Institute of Physiology, Zurich, Switzerland), rinsed, and incubated for 1 hour at room temperature with and FITC-coupled anti–rabbit IgG (1:500) and then observed on a confocal microscope (×40, Zeiss observer.Z1, LSM710).

Two serial sections of human kidney biopsies were used for ASIC2 and AQP2 (a marker of the distal nephron) labeling. ASIC labeling was realized as for isolated CCDs. For AQP2, after blocking, slides were incubated with anti-AQP2 antibody (sc709882, 1:500, Santa Cruz Biotechnology) for 1 hour at room temperature, rinsed, and incubated with TRITC-conjugated anti–rabbit IgG (1:500) for 1 hour at room temperature.

### Albumin immunostaining.

Rat kidney sections (8 μm) fixed with 4% paraformaldehyde and included in OCT were transferred to Superfrost Gold Glass slides, rinsed with PBS, incubated for 20 minutes at room temperature in 100 mM glycine, permeabilized for 30 s with 0.1% triton, and incubated for 5 minutes in SDS 1% for antigen retrieval. After blocking, slides were incubated with anti-AE1 antibody (1:500; a gift from C. Wagner, Institute of Physiology, Zurich, Switzerland) for 1 hour at room temperature, rinsed, and incubated with TRITC-coupled anti–rabbit IgG (1:500) for 1 hour at room temperature. After rinsing, anti-albumin antibody FITC-coupled was added (F0117, 1:500, DAKO).

### Quantification of phospho-ERK.

Cryosections (5 μm) of rat kidney fixed with 4% paraformaldehyde were transferred to Superfrost Gold Glass slides, rinsed with PBS at 4°C, and incubated for 10 minutes at 94°C in citrate buffer pH 6.0. After blocking with donkey serum (10%, for 30 minutes at room temperature), slides were incubated overnight at 4°C with anti-AQP2 antibody (sc-9882, 1:400, Santa Cruz Biotechnology) and anti–P-p44/42 MAPK (T202/Y204 antibody; Cell Signaling Technology, 9101S) and diluted at 1:400 each in PBS containing 5% milk and 0.01% Tween. Slides were rinsed 3 times for 5 minutes in PBS–Tween 0.01% and then incubated for 1 hour at room temperature with donkey fluor 488–conjugated anti–goat IgG (sc-362255, 1:500, Santa Cruz Biotechnology) and donkey Alexa Fluor 555–conjugated anti–rabbit IgG (02/2016, 1:2000, Life Technology) and DAPI 1:2000. After rinsing and mounting in Dako Glycergel mounting medium, slides were observed on a confocal microscope (×40, Zeiss observer.Z1, LSM710). Images were analyzed by NIH ImageJ software. The phospho-ERK fluorescence (red) in cross sections of AQP2^+^ tubules (green) was measured and expressed as a function of the tissue area of the cross section. Tubule sections (15–20) were analyzed in each animal, and the mean value was normalized to that of a same CC control WT rat analyzed in each experiment.

### Statistics.

Results are expressed as mean ± SEM from several animals. Comparison between groups was performed by either 2-tailed paired *t* test or unpaired *t* test or by variance analysis (1-way ANOVA) followed by post hoc multiple comparison test (Tukey’s test), as appropriate. A *P* value of less than 0.05 was considered significant.

### Study approval.

The animals were kept at Centre d’Explorations Fonctionnelles of the Cordeliers Research Center (CEF; agreement no. B75-06-12). All experimental protocols were performed in accordance with the institutional guidelines and the recommendations for the care and use of laboratory animals put forward by the Directive 2010/63/EU revising Directive 86/609/EEC on the protection of animals used for scientific purposes, which are the equivalent of the ARRIVE guidelines, and were approved by the Charles Darwin Ethic Committee (Ce5-2012-041). For the oocytes, the procedures for anesthesia and surgery were approved by the Paris-Descartes Ethic Committee for Animal Experiments (CEEA34.GP.011.12). Regarding the use of human samples, serial sections of frozen human kidney biopsies for RNA extraction or fixed human kidney biopsy for IHC were provided by the pathology department at Robert Debré Hospital. Informed consent of each patient was collected according to the French current regulation. cDNA samples from kidney biopsies of nonnephrotic patients were from a previously published series of experiments (38).

## Author contributions

AD, MF, AS, BV, and GC designed the project. AD wrote the first draft of the paper. MF, LC, CW, MG, LC, and AD carried out the animal experiments, biochemical, qPCR and immunolabeling analysis. MF, MP, and GD carried out the analysis of human samples. AS, GP, MK, and NB carried out electrophysiological analysis. GB and LC performed cloning of ASIC2b variant. IA and SR generated the rat ASIC2b^–/–^. CW, GC, and LC set up the colony of ASIC2b^–/–^ rats at the CEF animal facility and established the procedure for their genotyping. LM and PH carried out microperfusion on isolated tubules. All the authors discussed the results and commented on the manuscript.

## Supplementary Material

Supplemental data

## Figures and Tables

**Table 1:**
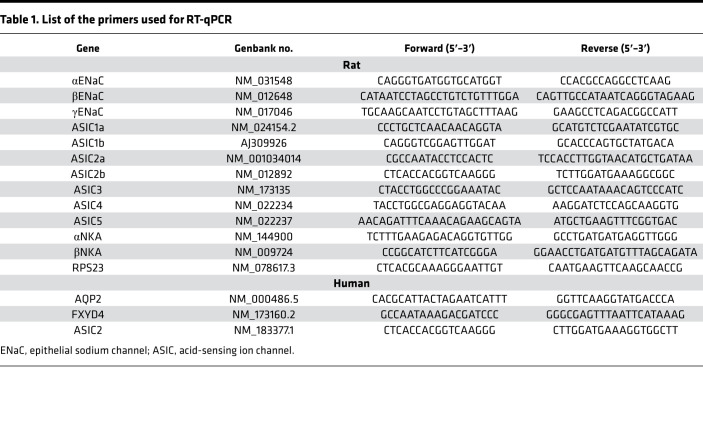
List of the primers used for RT-qPCR

**Table 2:**
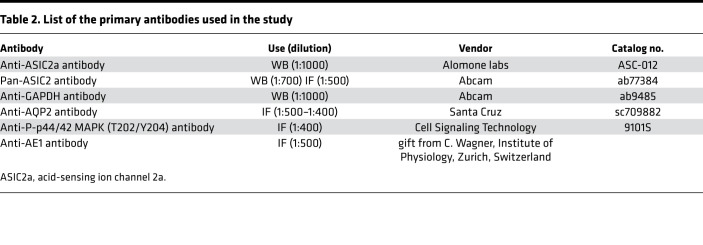
List of the primary antibodies used in the study

## Version 1 06/24/2021

In-Press Preview

## Version 2 08/09/2021

Electronic publication

## References

[1] Doucet A (2007). Molecular mechanism of edema formation in nephrotic syndrome: therapeutic implications. Pediatr Nephrol.

[2] Frenk S (1955). Experimental nephrotic syndrome induced in rats by aminonucleoside; renal lesions and body electrolyte composition. Proc Soc Exp Biol Med.

[3] Pedraza-Chaverri J (1990). Pathophysiology of experimental nephrotic syndrome induced by puromicyn aminonucleoside in rats. III. Effect of captopril, an angiotensin converting enzyme inhibitor, on proteinuria and sodium retention. Rev Invest Clin.

[4] Deschenes G (2001). Collecting duct is a site of sodium retention in PAN nephrosis: a rationale for amiloride therapy. J Am Soc Nephrol.

[5] Ichikawa I (1983). Role for intrarenal mechanisms in the impaired salt excretion of experimental nephrotic syndrome. J Clin Invest.

[6] Lourdel S (2005). Hyperaldosteronemia and activation of the epithelial sodium channel are not required for sodium retention in puromycin-induced nephrosis. J Am Soc Nephrol.

[7] Vande Walle JG (1995). Volume regulation in children with early relapse of minimal-change nephrosis with or without hypovolaemic symptoms. Lancet.

[8] de Seigneux S (2006). Increased expression but not targeting of ENaC in adrenalectomized rats with PAN-induced nephrotic syndrome. Am J Physiol Renal Physiol.

[9] Morla L (2013). Renal proteinase-activated receptor 2, a new actor in the control of blood pressure and plasma potassium level. J Biol Chem.

[10] Leviel F (2010). The Na^+^-dependent chloride-bicarbonate exchanger SLC4A8 mediates an electroneutral Na^+^ reabsorption process in the renal cortical collecting ducts of mice. J Clin Invest.

[11] Kellenberger S, Schild L (2015). International union of basic and clinical pharmacology. XCI. structure, function, and pharmacology of acid-sensing ion channels and the epithelial Na+ channel. Pharmacol Rev.

[12] Kellenberger S, Schild L (2002). Epithelial sodium channel/degenerin family of ion channels: a variety of functions for a shared structure. Physiol Rev.

[13] Udwan K (2016). Oxidative stress and nuclear factor kappaB (NF-kappaB) increase peritoneal filtration and contribute to ascites formation in nephrotic syndrome. J Biol Chem.

[14] Deval E (2010). Acid-sensing ion channels (ASICs): pharmacology and implication in pain. Pharmacol Ther.

[15] Lingueglia E (2007). Acid-sensing ion channels in sensory perception. J Biol Chem.

[16] Ugawa S (2003). Amiloride-insensitive currents of the acid-sensing ion channel-2a (ASIC2a)/ASIC2b heteromeric sour-taste receptor channel. J Neurosci.

[17] Dizin E (2013). Albuminuria induces a proinflammatory and profibrotic response in cortical collecting ducts via the 24p3 receptor. Am J Physiol Renal Physiol.

[18] Fila M (2011). Inhibition of K+ secretion in the distal nephron in nephrotic syndrome: possible role of albuminuria. J Physiol.

[19] Chambrey R (2013). Renal intercalated cells are rather energized by a proton than a sodium pump. Proc Natl Acad Sci U S A.

[20] Morla L (2016). The renal cortical collecting duct: a secreting epithelium?. J Physiol.

[21] Edwards A, Crambert G (2017). Versatility of NaCl transport mechanisms in the cortical collecting duct. Am J Physiol Renal Physiol.

[22] Dawson RJ (2012). Structure of the acid-sensing ion channel 1 in complex with the gating modifier Psalmotoxin 1. Nat Commun.

[23] Jasti J (2007). Structure of acid-sensing ion channel 1 at 1.9 a resolution and low pH. Nature.

[24] Canessa CM (1994). Amiloride-sensitive epithelial Na+ channel is made of three homologous subunits. Nature.

[25] DuBose TD (1979). , et al. Micropuncture determination of pH, PCO2, and total CO2 concentration in accessible structures of the rat renal cortex. J Clin Invest.

[26] Weinstein AM (2010). A mathematical model of rat ascending Henle limb. III. Tubular function. Am J Physiol Renal Physiol.

[27] Cadiou H (2007). Modulation of acid-sensing ion channel activity by nitric oxide. J Neurosci.

[28] Smith ES (2007). Arachidonic acid potentiates acid-sensing ion channels in rat sensory neurons by a direct action. Neuroscience.

[29] Li WG (2010). ASIC3 channels integrate agmatine and multiple inflammatory signals through the nonproton ligand sensing domain. Mol Pain.

[30] Yu Y (2010). A nonproton ligand sensor in the acid-sensing ion channel. Neuron.

[31] Passero CJ (2008). Plasmin activates epithelial Na+ channels by cleaving the gamma subunit. J Biol Chem.

[32] Svenningsen P (2009). Plasmin in nephrotic urine activates the epithelial sodium channel. J Am Soc Nephrol.

[33] Falin R (2005). A role for ERK1/2 in EGF- and ATP-dependent regulation of amiloride-sensitive sodium absorption. Am J Physiol Cell Physiol.

[34] Niisato N (2012). Hypotonic stress upregulates β- and γ-ENaC expression through suppression of ERK by inducing MKP-1. Am J Physiol Renal Physiol.

[35] Trac PT (2017). Alveolar nonselective channels are ASIC1a/α-ENaC channels and contribute to AFC. Am J Physiol Lung Cell Mol Physiol.

[36] Deschenes G, Doucet A (2000). Collecting duct (Na+/K+)-ATPase activity is correlated with urinary sodium excretion in rat nephrotic syndromes. J Am Soc Nephrol.

[37] Menoret S (2015). Homology-directed repair in rodent zygotes using Cas9 and TALEN engineered proteins. Sci Rep.

[38] Disset A (2009). Tissue compartment analysis for biomarker discovery by gene expression profiling. PLoS One.

